# Supplementary material for: Causal Relationship between Aging and Anorexia Nervosa: A White-Matter-Microstructure-Mediated Mendelian Randomization Analysis
Source: Biomedicines. 2024 Aug 16;12(8):1874. doi: 10.3390/biomedicines12081874 (PMC11351342; doi:10.3390/biomedicines12081874)
Supplement: Supplementary file 1 [file biomedicines-12-01874-s001.zip › Supplementary Figure/Figure S4/Singe SNP plot of MR analysis for TL to ubm-b-1643.pdf]

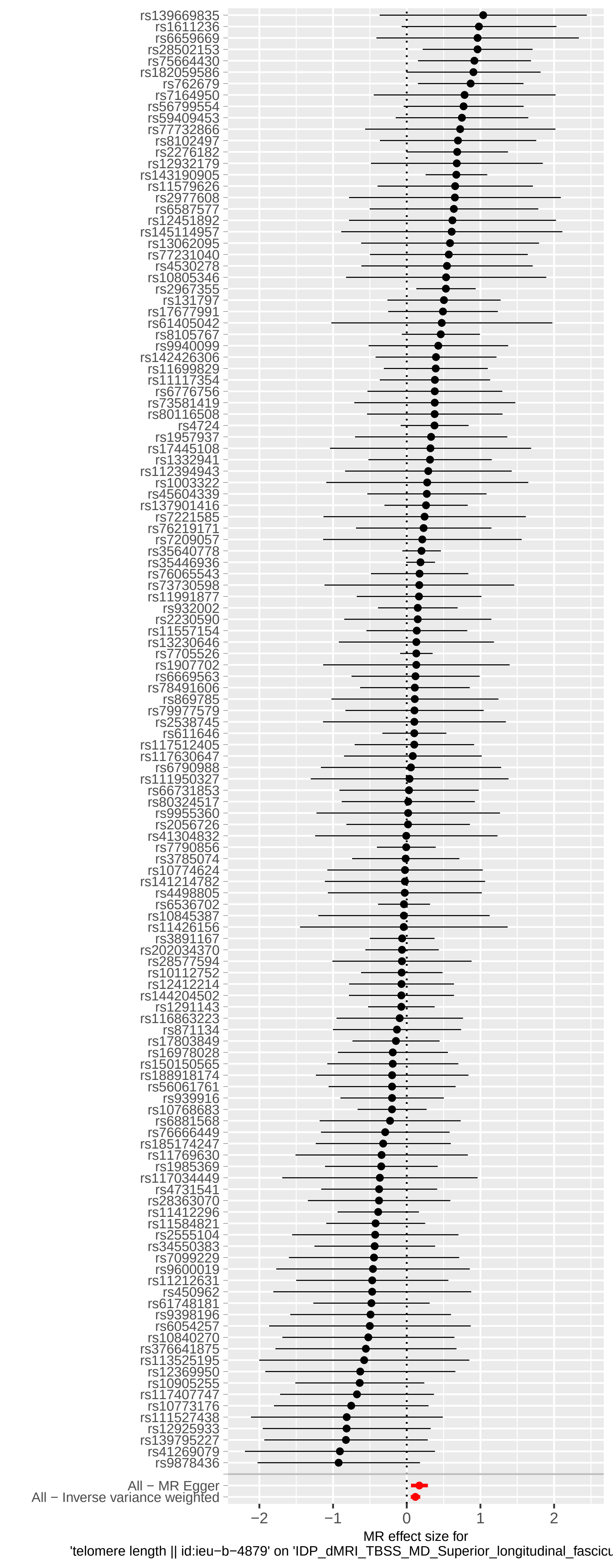
rs139669835
rs1611236
rs6659669
rs28502153
rs75664430
rs182059586
rs762679
rs7164950
rs56799554
rs59409453
rs77732866
rs8102497
rs2276182
rs12932179
rs143190905
rs11579626
rs2977608
rs6587577
rs12451892
rs145114957
rs13062095
rs77231040
rs4530278
rs10805346
rs2967355
rs131797
rs17677991
rs61405042
rs8105767
rs9940099
rs142426306
rs11699829
rs11117354
rs6776756
rs73581419
rs80116508
rs4724
rs1957937
rs17445108
rs1332941
rs112394943
rs1003322
rs45604339
rs137901416
rs7221585
rs76219171
rs7209057
rs35640778
rs35446936
rs76065543
rs73730598
rs11991877
rs932002
rs2230590
rs11557154
rs13230646
rs7705526
rs1907702
rs6669563
rs78491606
rs869785
rs79977579
rs2538745
rs611646
rs117512405
rs117630647
rs6790988
rs111950327
rs66731853
rs80324517
rs9955360
rs2056726
rs41304832
rs7790856
rs3785074
rs10774624
rs141214782
rs4498805
rs6536702
rs10845387
rs11426156
rs3891167
rs202034370
rs28577594
rs10112752
rs12412214
rs144204502
rs1291143
rs116863223
rs871134
rs17803849
rs16978028
rs150150565
rs188918174
rs56061761
rs939916
rs10768683
rs6881568
rs76666449
rs185174247
rs11769630
rs1985369
rs117034449
rs4731541
rs28363070
rs11412296
rs11584821
rs2555104
rs34550383
rs7099229
rs9600019
rs11212631
rs450962
rs61748181
rs9398196
rs6054257
rs10840270
rs376641875
rs113525195
rs12369950
rs10905255
rs117407747
rs10773176
rs111527438
rs12925933
rs139795227
rs41269079
rs9878436
All − MR Egger
All − Inverse variance weighted
−2
−1
0
1
2
MR effect size for
'telomere length || id:ieu−b−4879' on 'IDP_dMRI_TBSS_MD_Superior_longitudinal_fascicu
